# Aging and Death in an Organism That Reproduces by Morphologically Symmetric Division

**DOI:** 10.1371/journal.pbio.0030045

**Published:** 2005-02-01

**Authors:** Eric J Stewart, Richard Madden, Gregory Paul, François Taddei

**Affiliations:** **1**Inserm, U571ParisFrance; **2**UniversitéParisFrance; **3**Institut des Hautes Etudes Scientifique, Le Bois-MarieBures-sur-YvetteFrance; University of Newcastle upon TyneUnited Kingdom

## Abstract

In macroscopic organisms, aging is often obvious; in single-celled organisms, where there is the greatest potential to identify the molecular mechanisms involved, identifying and quantifying aging is harder. The primary results in this area have come from organisms that share the traits of a visibly asymmetric division and an identifiable juvenile phase. As reproductive aging must require a differential distribution of aged and young components between parent and offspring, it has been postulated that organisms without these traits do not age, thus exhibiting functional immortality. Through automated time-lapse microscopy, we followed repeated cycles of reproduction by individual cells of the model organism *Escherichia coli,* which reproduces without a juvenile phase and with an apparently symmetric division. We show that the cell that inherits the old pole exhibits a diminished growth rate, decreased offspring production, and an increased incidence of death. We conclude that the two supposedly identical cells produced during cell division are functionally asymmetric; the old pole cell should be considered an aging parent repeatedly producing rejuvenated offspring. These results suggest that no life strategy is immune to the effects of aging, and therefore immortality may be either too costly or mechanistically impossible in natural organisms.

## Abstract 2

Detailed time lapse photography reveals that organisms that divide symmetrically, such as the bacterium E. coli, can indeed age and consequently that no organism is immune to mortality

## Introduction

That populations survive indefinitely while individuals grow old and die requires that young offspring be produced at the expense of old parents, and this has classically been explained in terms of an immortal germ line passing through a transient and disposable soma, or body [1]. However, with the discovery of aging in single-celled organisms with no clear separation of these two constituents, it has been proposed that reproduction by asymmetric division is a prerequisite for aging [2] and that organisms that reproduce without a distinction between parent and offspring do not age, thus exhibiting functional immortality [3,4].

One difficulty with this distinction is that an asymmetry sufficient for aging may not necessarily result in a visible difference during cell division. Therefore, what may be a more useful common feature of the organisms that have currently been found to age is the presence of a juvenile phase (developmental asymmetry) in their life cycle. Juvenile cells are either smaller or undifferentiated and must go through a period of growth or differentiation before becoming capable of reproducing. This is similar to the idea that the critical requirement for aging in unicellular organisms is the presence of a parent cell that provides for a smaller offspring cell [2]. Consistent with this, the primary single-celled organisms that have been shown to age (the yeast Saccharomyces cerevisiae and the bacterium Caulobacter crescentus) share the traits of a visibly asymmetric division and an identifiable juvenile phase [3,5,6]. The report of aging in the binary fission yeast Schizosaccharomyces pombe has reinforced these apparent characteristics of aging organisms. The same study that found indications of aging in this organism also found that, contrary to expectations, its cell division process was visibly asymmetric, resulting in enlarged mother cells after only a few divisions, possibly indicating that S. pombe development is similar to that of budding yeast [7].

**Figure 1:**
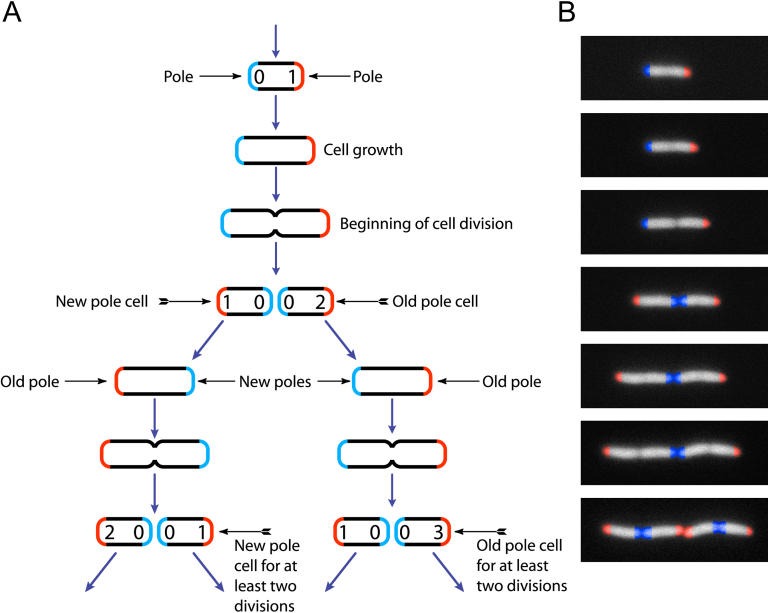
The Life Cycle of E. coli During cell division, two new poles are formed, one in each of the progeny cells (new poles, shown in blue). The other ends of those cells were formed during a previous division (old poles, shown in red). (A) The number of divisions since each pole was formed is indicated by the number inside the pole. Using the number of divisions since the older pole of each cell was formed, it is possible to assign an age in divisions to that cell, as indicated. Similarly, cells that consecutively divided as a new pole are assigned a new pole age, based on the current, consecutive divisions as a new pole cell. (B) Time-lapse images of growing cells corresponding to the stages in (A). False color has been added to identify the poles.

Here we look for evidence of aging in Escherichia coli, that is, in cells that do undergo a morphologically symmetrical division and do not exhibit a juvenile phase, in order to determine if such organisms age. Previous studies of senescence in E. coli have mostly focused on the loss of viability over time during nutrient depletion (conditional senescence) [4]. However, these studies do not address aging in terms of parent and offspring (reproductive life span), nor do they address growing cells that are not under conditions of starvation. To identify reproductive life-span effects, it is necessary to follow individual cells as they grow and reproduce over time. The bacterium E. coli grows in the form of a rod, which reproduces by dividing in the middle. One new end (or pole) per progeny cell is produced during this division event (Figure 1). Therefore, one of the ends of each cell has just been created during division (termed the new pole), and one is pre-existing from a previous division (termed the old pole). Old poles can exist for many divisions, and if cells are followed over time, an age in divisions can be assigned to each pole, and hence to each cell. While experiments following the partitioning of cell constituents have found uniform distributions of DNA and lipids in daughter cells [8], it is known that components of the cell wall turn over slowly and are conserved in the poles where they are formed [9]. More generally, any cell constituent with limited diffusion and a long half-life may be expected to accumulate at the old pole, so there may exist a physiological (rather than morphological) asymmetry between the old and new poles. Limited by the manual measurements required, individual cells growing under the microscope have been followed in the past for a small number of divisions [10,11,12]. The only difference between poles was seen in a single experiment that indicated that old pole cells might divide later than their new pole siblings, hinting at a possible inequality between these cells [13]. To determine if E. coli experiences aging related to the inheritance of the old pole, we followed individual exponentially growing cells in an automated fluorescence microscopy system through up to nine generations of growth and reproduction, measuring the physical parameters of each cell over time. We present conclusive evidence for aging in the old pole cell, including cumulatively slowed growth, less offspring biomass production, and an increased probability of death.

## Results

**Figure 2:**
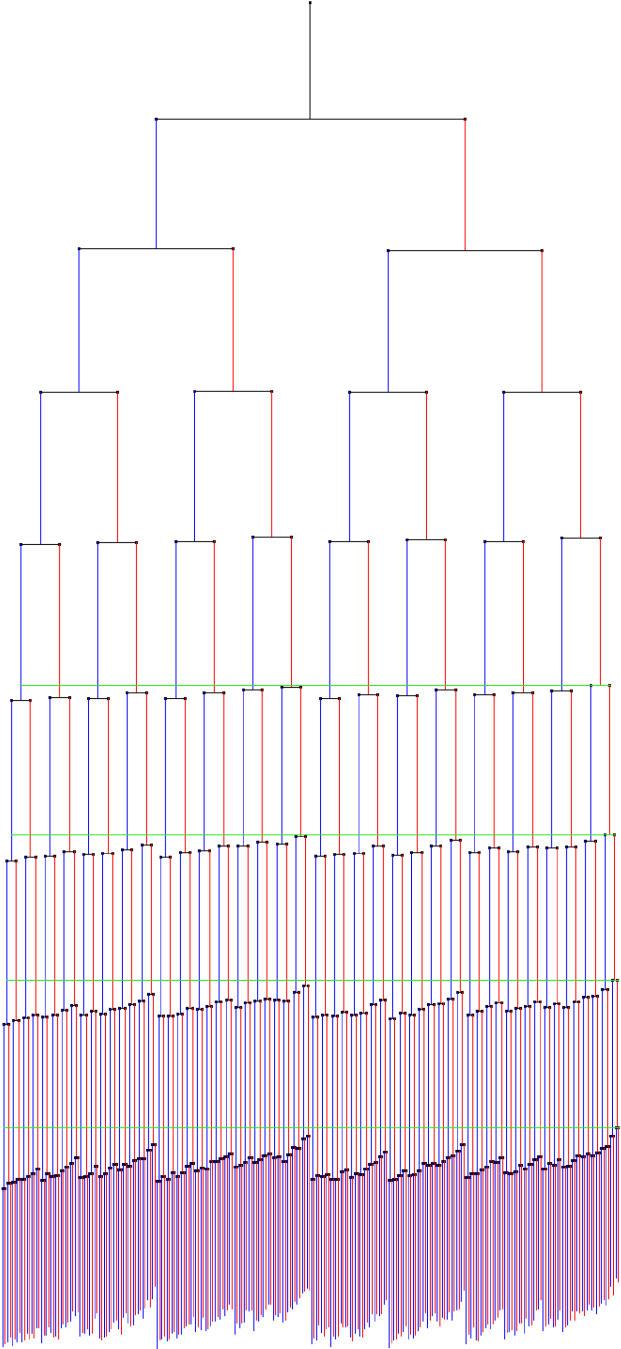
Average Lineage Showing Old Pole Effect on Growth Rate The first division in the microcolonies is not represented, as the identity of the poles is not known until after one division (hence each initial cell gives rise to two lineages that are tracked separately, and subsequently combined from all films to create the single average lineage shown here). The lengths of the lines connecting cells to their progeny are proportional to the average growth rate of that cell; a longer line represents a higher growth rate for that cell. At each division, the cell inheriting the old pole is placed on the right side of the division pair, and shown in red, while new poles are placed on the left side of each pair, and shown in blue (note that this choice of orientation is not the same as that of Figure 1, to compare more easily old and new pole lineages). Because the position of the start of the growth line for each new generation is dependent on the generations that preceded it, the difference in growth rates is cumulative. Green lines indicate the point at which the first cell divides in the last four generations. Nine generations from 94 films encompassing 35,049 cells are included in this tree. The average growth rate of all the cells corresponds to a doubling time of 28.2+/−0.1 min. The data used to generate the average lineage are provided in Dataset S1.

Cells were grown from one cell into a monolayer microcolony that contained up to 500 cells, and time-lapse images (see Video S1 for an example film) were analyzed with custom automated software designed for this purpose. We followed 94 such colonies, resulting in the complete record from division to division, of 35,049 cells. As the history and physical parameters of each cell in the microcolony are known, and the identity of each pole is tracked, the complete lineage can be determined. The resulting lineages from each film were averaged by each unique cell position within the lineage. This can be represented as a single, bifurcating tree, where each branch point is an average cell for that position in the lineage, and the length of the lines connecting cells to their progeny are proportional to the growth rate of the cell (Figure 2). At each division in the tree, the cell inheriting the old pole of the progenitor cell is represented on the right branch of the sibling pair (in red), while the cell inheriting the new pole is on the left branch (in blue).

The pattern of fast and slow growth rates in this average lineage gives striking evidence for reproductive asymmetry between the progeny cells, as the cells that show a cumulatively slowed rate of growth (shorter lines) are those cells that have more often inherited an old pole during their ancestry. To verify that this pattern in the average lineage is actually due to a difference in growth rate between new and old pole cells, we performed a pairwise comparison of every set of sister cells that was produced at the eighth generation in each of the films. As sister cells share temporal and spatial surroundings, this comparison controls for potential environmental variation within the microcolony. The comparison (two-tailed *t*-test) includes cells of all division ages and shows that the average growth rate of the old pole progeny cell is 2.2% (+/−0.1%) slower than that of the new pole cell. This analysis, performed on 7,953 cell pairs, conclusively demonstrates (*p* < 0.00001, *t* = 14.40, *df* = 7952) that the cell that inherits the old pole grows slower than the new pole cell produced in the same division. Two factors from this same dataset demonstrate the lack of a juvenile phase in *E. coli.* First, comparison of the progeny cells shows that the new pole cell is slightly larger on average (0.9+/−0.1%; *p* < 0.00001, *t* = 5.62, *df* = 7952) than the old pole cell (the contrary would be expected in the presence of a juvenile phase). Second, the young pole cell is marginally more likely to divide sooner than the old pole cell (in about 15% of the cases cells divide within the same 2-min time point; of those where the two cells divide in different time points, 54% of the time the new pole cell divides first [significant; *p* < 0.00001, *t* = 5.02, *df* = 4812]), which is also not consistent with a phase where the young cell must grow or differentiate before reproduction. These differences are consistent for all generations during steady-state growth (data not shown). Therefore, while a juvenile phase is absent, there is a consistent functional asymmetry between the two progeny cells that is disadvantageous to the old pole.

**Figure 3:**
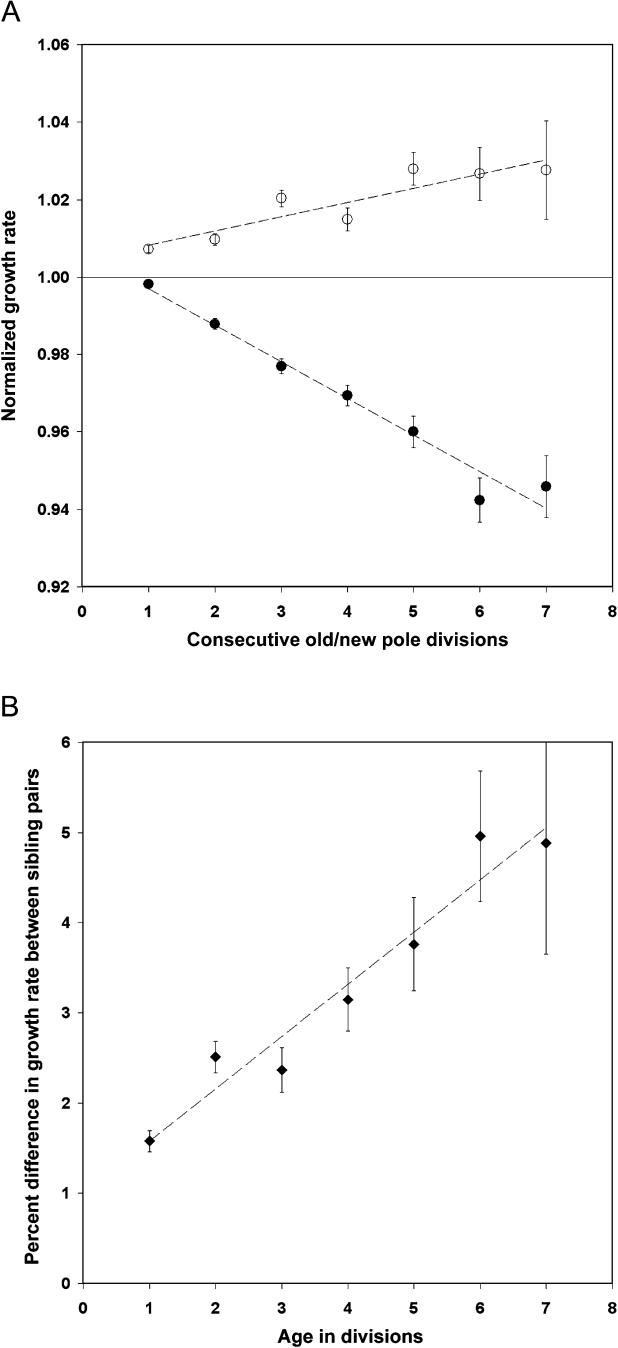
The Effects of Consecutive Divisions as an Old or New Pole on Growth Rate (A) The cellular growth rate, represented on the y-axis, is normalized to the growth rate of all cells from the same generation and geography in each film. On the x-axis consecutive divisions are seen as either a new pole (open circles), showing rejuvenation, or an old pole (closed circles), showing aging. Cells represented at each point: new pole divisions 1–7: 7*,*730; 3*,*911; 1,956; 984; 465; 211; 89; old pole divisions 1–7: 4,687; 3,833; 1,933; 956; 465; 213; 75. (B) Pair comparison of the growth rates of sibling cells. The division age of the old pole sibling (the mother cell) is shown on the x-axis. The percentage difference between the growth rate of the new pole sibling (the daughter cell) and this cell is shown on the y-axis. A positive difference corresponds to a faster growth rate for the new pole cell. Cell pairs represented at each point, ages 1–7: 9,722; 4,824; 2,409; 1,202; 601; 282; 127. In both graphs, cells are from all 94 films. The error bars represent the standard error of the mean. The old and new pole growth rates in (A) and the pair differences in (B) are fitted to a line to show the trend; however, the actual progressions may not be linear (*R* ^2^ old poles = 0.97, new poles = 0.83, pair difference = 0.94).

Each cell is defined not just by its preceding division, however, but also by all previously recorded divisions, back to the initial cell in the analysis. Therefore, each old pole cell can be categorized by the number of consecutive, final old pole divisions that occurred to produce the current cell (thus giving the age in divisions of its old pole). Equivalently, each new pole cell can be assigned a number of divisions that it sequentially divided as the new pole cell. Comparing these values with the growth rates of the cells, we find that the older the old pole of a cell is, the slower the growth rate of that cell, while cells with more consecutive new pole divisions exhibit increasing growth rates (Figure 3A). Furthermore, a pairwise comparison shows that the difference in the growth rate between the old pole sibling (the mother cell) and the new pole sibling (daughter cell) increases with the increasing age of the mother (Figure 3B). Therefore, the difference between pairs of progeny cells, as well as the pattern seen in the average lineage, is not only due to a decrease in growth rate of cells that have inherited the old pole, but also to an increase in the growth rate (for at least three divisions; subsequent divisions do not detectably improve) of cells that have repeatedly inherited the new pole.

To determine the longer-term effect of inheriting the old pole, we performed a second pairwise analysis, comparing the total length of offspring produced by sister cells from the fifth generation until the end of tracking (this generation was chosen as each cell has the opportunity to progress through about three divisions). As the bacteria are rod shaped, the total length of cells produced is proportional to the biomass of the offspring. The results show that old pole cells produce less offspring biomass compared to their new pole sisters (3.1+/−0.3% less, *p* < 0.00001, *t* = 9.29, *df* = 1565). Therefore, it appears that the slower growth rate of the old pole cells also results in a longer-term decreased ability to produce offspring biomass. Another long-term effect of aging is the probability of survival of the organism over time. During the growth of the microcolonies, sixteen cells were observed to cease growing; these cells never resumed growth during the course of the experiment. We have defined these cells as potentially dead cells and have analyzed their locations in the lineages. While these apparent deaths may ultimately be due to stochastic events, they show a statistically significant bias (*p* = 0.01; see Materials and Methods) toward increased divisions spent as an old pole (over the total observation history). This observation is consistent with the hypothesis that aged cells are more susceptible to harmful events and/or less likely to survive them. It is unlikely that these cells represent a growth arrested “persister” state, as it has recently been demonstrated that persister cells that arise during exponential growth occur at a frequency of approximately 1.2 × 10^−6^ [14]; the appearance of apparently dead cells in our study (about 4.6 × 10^−4^) is almost 400 times more frequent.

## Discussion

We find that the old pole is a significant marker for multiple phenotypes associated with aging, namely, decreased metabolic efficiency (reduced growth rate), reduced offspring biomass production, and an increased chance of death. Thus, *E. coli,* an organism with a morphologically symmetrical division, no juvenile phase, and no identified separation between germ line and soma, is susceptible to aging. Unlike the process of clonal senescence [15], where an entire population progressively declines in fitness, here the life potential of the lineage is continually renewed through young offspring cells (the process of rejuvenation) that are produced at the expense of aged parent cells.

That the two cells are not equal, despite appearances, indicates a functional asymmetry that may be explained by a number of mechanisms, such as those that result in the polar localization of cell components [16,17]. In the simplest example, any component localized to the cell poles will have more time to accumulate in an old pole than in a young one. However, the pole effect may involve more active processes (such as differential turnover or accumulation) because, in addition to the aging effect of the parent, new pole cells show a concomitant increase in their growth and reproduction over several divisions. This result is not without precedent in aging organisms. In the budding yeast S. cerevisiae, the daughter cell of a young mother cell has a greater reproductive life span compared to the daughter cell of an old mother cell [18]. A likely explanation for this effect in yeast is the loss of segregation control of detrimental cellular components, such as extrachromosomal rDNA circles [19], and possibly damaged mitochondria as well [20]. Additionally, in fruit flies (Drosophila melanogaster), several generations of daughters are required to recover from the effects of chromosome damage [21]. In our study, the new pole cells can apparently benefit from the preferential distribution of cellular components at the expense of the old pole cell for at least three divisions. After further divisions as a new pole cell, it is not clear that there is a continuing benefit to the cell. The observation that an optimum is not reached in a single division implies that the mechanism responsible for an age-related bias in cell component inheritance is not absolute; repeated rounds of sorting continue to improve the condition of new pole cells.

On the other hand, the observed rate of decline in old pole cells (about 1% of the initial value per division) would result in a complete cessation of growth after about 100 divisions. While the behavior of cells with more than seven old pole divisions cannot be determined from these data, it is interesting to note that the observed rate of decline in offspring production in C. crescentus (a bacterium with an obviously asymmetric division and a developmentally significant juvenile phase) is of about the same magnitude as the decline in growth rate measured here [3]. In S. cerevisiae, the rate of offspring production also declines, resulting in cell cycle times that are as much as five times longer for mother cells that have divided 30 times than for young cells. This is an accelerating decline, however, and there is no detectable difference in cell cycle time for the first ten divisions [22].

Our results show that a juvenile phase is not required for the process of aging any more than the presence of a germ line or a visibly asymmetric division is. In contrast, we demonstrate the presence of a physiological asymmetry in *E. coli,* which is essential for the process of aging. The cost of the aging process in lost growth to the population under these conditions is about 2%. In competition, these cells would be rapidly displaced by competitors that did not age, but only if the cost of avoiding senescence were not equal or greater. It has been proposed that one such cost is the rigorous level of maintenance and repair that would be required to prevent the decline and eventual extinction of a perfectly symmetrical organism [23]. The physiological asymmetry during division may therefore represent the disposal by preferential partitioning of cellular damage that is expensive or impossible to repair. Concerning the evolutionary origin of the aging process seen here, it is possible that an asymmetry of division existed before aging appeared as a life history trait in these cells, and that such an inequality may have therefore allowed (or forced) aging to occur. However, as we have detected this asymmetry solely through phenotypes that can be linked to aging, it is equally possible that the necessity of aging is itself the evolutionary cause of the asymmetry. If this latter explanation is found to be correct, then the occurrence of aging in a single-celled organism that is apparently meticulously symmetric otherwise may indicate that it is either not cost effective in general to produce an immortal life form, or it is impossible to achieve perfect molecular maintenance through natural selection.

The use of the model organism E. coli provides an excellent genetic platform for studying the fundamental mechanisms of cellular aging and may provide insight into the costs and evolutionary roots of repair, maintenance, and longevity. Simple model organisms such as yeast and nematodes have already proven their value in identifying evolutionarily conserved pathways that regulate life span in higher organisms [24,25]. As has been seen with many fundamental life functions, it may be that the primary processes involved in aging in E. coli will also be conserved in other forms of life.

## Materials and Methods

### 

#### Strain and growth conditions

The sequenced wild-type strain of *E. coli,* MG1655 [26], was modified to express the gene encoding yellow fluorescent protein under the control of the lactose operon repressor and the Pl promoter of lambda phage (gene construct from M. Elowitz [27]). Cells were inoculated onto microscope cavity slides from exponentially growing liquid cultures, such that the colonies and cells grew exponentially in a single plane on the surface of a solid matrix of LB-agarose (NaCl concentration of 5 g/l, supplied by SdS, Peypin, France; other components were DIFCO from Becton Dickinson, Franklin Lakes, New Jersey, United States; agarose from QA-Agarose, Qbiogene, Irvine, California, United States; plus 1 mM IPTG from Qbiogene). The slide cavities were sealed with silicone grease and contained sufficient oxygen and nutrients to allow undiminished growth and fluorescence for the length of the experiment. The slides were incubated in a temperature-controlled (Cube and Box incubation system, Life Imaging Services, Reinach, Switzerland) automated microscope (Zeiss 200M; Zeiss, Jena, Germany) at 30 °C for up to 6 h. The entire microscope was contained within the incubator, eliminating temperature gradients. These conditions resulted in an excess of nutrients and a protected environment without external causes of cell mortality.

#### Microscopy

Up to seven fields containing one to four cells each were manually identified at the start of the experiment, and stored in the MetaMorph microscope control software (Universal Imaging, Downingtown, Pennsylvania, United States). Fluorescent images were recorded at each field with time points taken generally every 4 min for the first 160 min, then every 2 min for the remaining time. A subset of six colonies was recorded every 40 s for improved time resolution. The excitation light did not affect the cellular growth rate (data not shown). Images were taken with a CoolSNAP HQ (Princeton Instruments, Trenton, New Jersey, United States) at 100X magnification; the resulting images have a spatial dimension of 0.064 μ per pixel. Excitation light (480 to 520 nm) was limited to 5% of the output of a 100-W Hg vapor lamp, with an exposure of 2 s. Emission wavelengths were 505 to 565 nm (filters from Chroma, Rockingham, Vermont, United States). See Video S1 for a sample film.

#### Image analysis

The custom analysis software (BHV) was developed by integration of open-source software under a central shell scripted in Python [28]. For pixel-intensive operations, C routines were written and linked to the Python shell. To segment the cells in the images, local minima were identified to outline the cells, then eroded to remove remaining connections, and dilated back to size. The measurement process calculated the second moments of the cell's pixel intensity/location distribution and matched it to a rectangle with the same parameters. Long, curved cells that could not be approximated to a rectangle were measured manually, using tools built into the software. Frames were then compared at successive time points, and cells were identified from frame to frame by their overlap with the previous frame, taking into account predicted cell movement due to growth of the colony. This process tracked 80% of the cells into the ninth generation without manual intervention, measuring approximations to their length, width, fluorescence intensity, orientation, geographical location within the colony, and the complete lineage history of each cell. The rate of change of each of these parameters was calculated, with the growth rate being represented by an exponential fit to length over time. Length is proportional to cell mass, as cells did not increase in width during growth. The limits to the cell-tracking process were expansion of the colony beyond the image frame and the formation of multiple layers of cells. Manual measurements were used to correct tracking errors, which allowed us to produce datasets for colonies that are up to 100% tracked. Therefore, the complete history of each cell, including how many times it divided and its relationship with the other cells in the lineage, was unambiguously determined.

#### Statistical analysis

We verified that our results are not the effect of bias unrelated to pole age in two ways. First, we compared pairs of daughters of the same cell using a pairwise *t*-test. Since the sibling cells occupy similar points in space and time, this eliminates influences coming from overall changes in the colony growth rate or potential variations in environment across the colony. Second, we created control datasets (data not shown) from the colonies in which all the properties of the cells and lineages are preserved, with the exception of pole identity, which is randomly assigned at each division. In this case, we tested the null hypothesis that the pole age of a cell is not a factor in cell physiology by comparing the observed result with the expected normal binomial distribution derived from the null hypothesis. In both cases, we determined from these tests the probability that the old pole effect is due to random fluctuations, expressed as a *p*-value. We determined if the apparently dead cells were biased toward divisions as old pole cells by examining their complete recorded division histories, which describe how many times each cell divided as an old pole or new pole cell. We compared the average number of old pole divisions of the population of 16 dead cells (mean = 3.44) with the distribution of averages (mean = 2.75, SD = 0.29) from 1,000,000 randomly generated sets of 16 cells with the same number of total divisions (total divisions = 6, 6, 4, 4, 7, 4, 5, 7, 8, 3, 6, 6, 7, 7, 6, 2). This yielded the one-tailed probability (the hypothesis was that these cells would be enriched in old pole divisions, based on our observations of growth rates) of such an old pole bias arising by chance. All error ranges in the text and figures represent the standard error of the mean. The values for Figure 3A were normalized by finding the mean growth rate of all cells that shared similar conditions and dividing the individual growth rates of each cell by this mean. Similar conditions were defined as being in the same colony, with the same number of generations since the start of the film, and either in the interior of the colony or on the border (border cells exhibited a slight artifact in size measurement due to light spread in the optics; both interior and exterior populations showed a similar old pole effect).

## Supporting Information

Dataset S1 Table of Data for the Average Lineage Shown in Figure 2
The lineage is represented as a series of letters, where “O” indicates a division as an old pole cell, and “N” a division as a new pole cell. The first cell in the tree is labeled “–.” The number of cells indicates how many individual cell growth rates comprise the average rate. At early points in the lineage, there are many more than 94 cells (the number of films), as each initial cell gives rise to two lineages (as it is not possible to assign old/new pole status to the initial cell), and some films start with more than one cell. The average growth rates approximately correspond to microns per minute.
(52 KB XLS).

Video S1 Film of Growing Microcolony This film shows 305 min (114 frames) of the growth of a microcolony condensed to 7 s. For the first 40 frames (approximately 3 s), images were taken every 4 min; for the remaining frames, images were taken every 2 min. The complete lineage history of the entire microcolony from the single initial cell in frame 1 to all 505 cells in frame 114 has been tracked and recorded, allowing pole ages to be assigned to every cell.
(286 KB WMV).

## References

[pbio-0030045-b01] Kirkwood TBL, Holliday R (1979). The evolution of ageing and longevity. Proc R Soc Lond B Biol Sci.

[pbio-0030045-b02] Partridge L, Barton NH (1993). Optimality, mutation and the evolution of ageing. Nature.

[pbio-0030045-b03] Ackermann M, Stearns SC, Jenal U (2003). Senescence in a bacterium with asymmetric division. Science.

[pbio-0030045-b04] Nystrom T (2002). Aging in bacteria. Curr Opin Microbiol.

[pbio-0030045-b05] Sinclair DA (2002). Paradigms and pitfalls of yeast longevity research. Mech Ageing Dev.

[pbio-0030045-b06] Jazwinski SM (2002). Growing old: Metabolic control and yeast aging. Annu Rev Microbiol.

[pbio-0030045-b07] Barker MG, Walmsley RM (1999). Replicative ageing in the fission yeast Schizosaccharomyces pombe. Yeast.

[pbio-0030045-b08] Lin ECC, Hirota Y, Jacob F (1971). On the process of cellular division in Escherichia coli. VI. Use of a methocel−autoradiographic method for the study of cellular division in Escherichia coli. J Bacteriol.

[pbio-0030045-b09] de Pedro MA, Quintela JC, Holtje JV, Schwarz H (1997). Murein segregation in Escherichia coli. J Bacteriol.

[pbio-0030045-b10] Rahn O (1931). A chemical explanation of the variability of the growth rate. J Gen Physiol.

[pbio-0030045-b11] Kubitschek HE (1962). Normal distribution of cell generation rate. Exp Cell Res.

[pbio-0030045-b12] Schaechter M, Williamson JP, Hood JR, Koch AL (1962). Growth, cell and nuclear divisions in some bacteria. J Gen Microbiol.

[pbio-0030045-b13] Powell EO, Errington FP (1963). Generation times of individual bacteria: Some corroborative measurements. J Gen Microbiol.

[pbio-0030045-b14] Balaban NQ, Merrin J, Chait R, Kowalik L, Leibler S (2004). Bacterial persistence as a phenotypic switch. Science.

[pbio-0030045-b15] Hayflick L, Moorhead PS (1961). The serial cultivation of human diploid cell strains. Exp Cell Res.

[pbio-0030045-b16] Shapiro L, McAdams HH, Losick R (2002). Generating and exploiting polarity in bacteria. Science.

[pbio-0030045-b17] Lybarger SR, Maddock JR (2001). Polarity in action: Asymmetric protein localization in bacteria. J Bacteriol.

[pbio-0030045-b18] Kennedy BK, Austriaco NR, Guarente L (1994). Daughter cells of Saccharomyces cerevisiae from old mothers display a reduced life span. J Cell Biol.

[pbio-0030045-b19] Sinclair DA, Guarente L (1997). Extrachromosomal rDNA circles—A cause of aging in yeast. Cell.

[pbio-0030045-b20] Lai CY, Jaruga E, Borghouts C, Jazwinski SM (2002). A mutation in the ATP2 gene abrogates the age asymmetry between mother and daughter cells of the yeast Saccharomyces cerevisiae. Genetics.

[pbio-0030045-b21] Bregliano JC, Laurençon A, Degroote F (1995). Evidence for an inducible repair−recombination system in the female germ line of Drosophila melanogaster. I. Induction by inhibitors of nucleotide synthesis and by gamma rays. Genetics.

[pbio-0030045-b22] Egilmez NK, Jazwinski SM (1989). Evidence for the involvement of a cytoplasmic factor in the aging of the yeast Saccharomyces cerevisiae. J Bacteriol.

[pbio-0030045-b23] Kirkwood TBL, Townsend CR, Calow P (1981). Repair and its evolution: Survival versus reproduction. Physiological ecology: An evolutionary approach to resource use.

[pbio-0030045-b24] Tissenbaum HA, Guarente L (2002). Model organisms as a guide to mammalian aging. Dev Cell.

[pbio-0030045-b25] Hekimi S, Guarente L (2003). Genetics and the specificity of the aging process. Science.

[pbio-0030045-b26] Blattner FR, Plunkett G, Bloch CA, Perna NT, Burland V (1997). The complete genome sequence of Escherichia coli K−12. Science.

[pbio-0030045-b27] Elowitz MB, Levine AJ, Siggia ED, Swain PS (2002). Stochastic gene expression in a single cell. Science.

[pbio-0030045-b28] Python Software Foundation (2001–2003). Python: Versions 2.1 to 2.3 [computer program].

